# Anticholinergic medication use and falls in Australian residential aged care: a retrospective multisite cohort study

**DOI:** 10.1007/s40520-025-03147-9

**Published:** 2025-08-27

**Authors:** Ying Xu, Magdalena Z. Raban, Ling Li, Amy D. Nguyen, S. Sandun Malpriya Silva, Guogui Huang, Gaston Arnolda, Johanna I. Westbrook, Nasir Wabe

**Affiliations:** 1 Center for Health Systems and Safety Research, Faculty of Medicine, Health and Human Sciences, Australian Institute of Health Innovation, Macquarie University, Level 6, 75 Talavera Road, North Ryde, Sydney, NSW 2109 Australia; 2 St Vincent’s Clinical Campus, UNSW Sydney, Sydney, Australia

**Keywords:** Residential aged care, Nursing homes, Long-term care, Anticholinergic, Falls, Medication management

## Abstract

**Background:**

Associations between anticholinergic load and falls remain understudied in residential aged care facilities (RACFs).

**Aims:**

To examine associations between anticholinergic load and falls in the first year after entry to an RACF.

**Methods:**

We aggregated routinely collected data from 27 RACFs in New South Wales, Australia. Anticholinergic load and falls were repeatedly measured for one year after residents first entered an RACF. Thirteen 28-day review periods were set. Associations between anticholinergic load in a review period and any falls in the next review period were examined, comprising 12 repeated measurements of associations. We included new residents aged ≥ 65 years, who entered an RACF between 1 July 2014 and 2 September 2021. Six scales were used: Anticholinergic Cognitive Burden (ACB), Anticholinergic Drug Scale (ADS), Anticholinergic Loading Scale (ALS), Anticholinergic Risk Scale (ARS), Chew’s list, and Clinician-rated Anticholinergic Score (CrAS). We used mixed-effect logistic regression models, adjusting for potential confounders. Facility was used as a cluster variable.

**Results:**

For the 2300 residents (67.7% females), there were steady increases in mean anticholinergic load from the first to the 12th review period. Per one-point higher anticholinergic load was associated with an increased risk of falls, adjusted odds ratios: 1.08 (95% confidence interval[CI] 1.04, 1.12) using ACB, 1.11 (95%CI 1.06, 1.15) using ADS, 1.15 (95%CI 1.10, 1.21) using ALS, 1.10 (95%CI 1.04, 1.17) using ARS, 1.18 (95%CI 1.09, 1.27) using Chew’s list, and 1.14 (95%CI 1.10, 1.19) using CrAS.

**Conclusion:**

Anticholinergic scales may be useful to inform falls prevention programs for new RACF residents.

**Supplementary Information:**

The online version contains supplementary material available at 10.1007/s40520-025-03147-9.

## Introduction

Medications with anticholinergic effects (anticholinergics) are widely used for common conditions such as Parkinson's disease, bradycardia, chronic obstructive pulmonary disease, depression, and urinary incontinence [1]. Older adults are susceptible to side effects from anticholinergics [2–4]. The central side effects of dizziness, headache, ataxia, and confusion are associated with increased risk of falls [5]. Changes in medication use often occur soon after residents enter residential aged care facilities (RACFs, also known as nursing homes). For instance, numbers of dispensed psychotropic [6], glucose-lowering [7], and any medications [8] have been shown to increase in the first few months after entry.

However, studies on longitudinal patterns of anticholinergic load are sparse, especially after RACFs entry [9, 10]. Meanwhile, upon entering RACFs, residents encounter new environmental hazards, which may elevate their risk of falling [11]. Further, previous studies reported relationships between anticholinergic load and falls, for example, in a population-based New Zealand study of those aged 65 years and over [9], and in the community-based UK Biobank cohort study [10]. Yet, there have been lacked information on falls not serious enough to cause hospitalization [9, 10]. This is a potentially important limitation as falls, even non-injurious, are related to fear of falling, reduced confidence and functional decline [12].

Investigation of rates of potentially inappropriate prescribing in primary care, with the most common involving anticholinergics, were found to be double among people with dementia compared to those without [13]. Different predictors of falls have been identified in a population-based survey among community-living older adults with and without dementia [14], but it remains uncertain whether the relationship between anticholinergics and falls differ for RACF residents with and without dementia. We aimed to examine associations between anticholinergic load and risks of falls in RACF residents, overall and separately for those with and without dementia, in the first year after their RACFs entry.

## Methods

### Study design, setting, data source, and ethics approval

We conducted a retrospective longitudinal study using routinely collected data from 27 RACFs managed by a large not-for-profit aged care provider in New South Wales, Australia. One RACF was in inner regional area and 26 were in major cities [15]. De-identified residents’ data were collected from the clinical and care management information systems. This includes residents’ year of birth, sex, entry and departure dates, health conditions recorded at entry, daily medication administration, and incident reports between 1 July 2014 and 31 August 2022. This study was undertaken as part of a larger program of research conducted in partnership with the aged care provider [16]. Ethics was approved by the Macquarie University Human Research Ethics Committee (Project ID: 12,513). In line with Australia's National Statement on Ethical Conduct in Human Research (2023), a waiver of consent was granted by the Macquarie University Human Research Ethics Committee.

### Study population

**Fig. 1:**
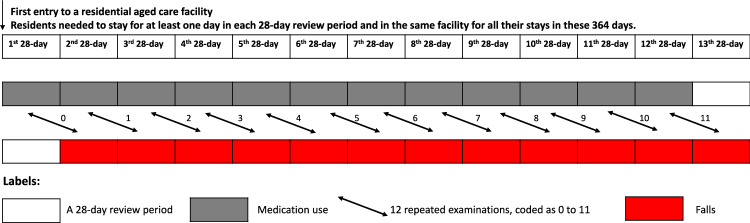
Study design

Residents aged ≥ 65 years old, who newly entered an RACF, for permanent or respite care, were followed for one year. Thirteen 28-day review periods were defined for each resident based on time since entry (Fig. 1). We adopted the 28-day to ensure identical numbers of weekdays and weekends. Our review frequency is set close to monthly, because recently raised anticholinergic load (within 30 days) has been related to greater risks of another important clinical endpoint, cardiovascular events [17]. Additionally, 30-day medication dispensing duration is common in Australia [18].

For a one-year (364 days) follow-up with 13 review periods (each with 28 days), residents’ first entry to an RACF needed to occur between 1 July 2014 and 2 September 2021. To further reduce heterogeneity, since residents who were in long-term care differed in their care needs and plan compared to respite residents who stayed for shorter periods, we restricted the study population to those who were resident in a single facility for at least one day in each review period for thirteen periods from entry.

### Anticholinergic load and other medication-related variables

Daily medication administration was documented in residents’ electronic clinical records. Medications prescribed as needed were counted, if administrated. The Anatomical Therapeutic Chemical (ATC) classification system was used to identify relevant medications.

We adopted six anticholinergic scales (Table S1) that were used in all three previous studies which had investigated relationships between falls and anticholinergic loads measured using multiple scales [9, 10, 19]. They were Anticholinergic Cognitive Burden (ACB) [20], Anticholinergic Drug Scale (ADS) [21], Anticholinergic Loading Scale (ALS) [22], Anticholinergic Risk Scale (ARS) [23], Chew’s list [24], and Clinician-rated Anticholinergic Score (CrAS) [25]. Medication administered were examined for the first 12 review periods since RACF entry (Fig. 1, review periods 1st-12th). In each review period, the anticholinergic load was calculated as the sum of anticholinergic ratings for all different medications administered, adopting each of the six scales.

Medications which increase falls risk, but do not contribute to anticholinergic load (not listed in the corresponding scale), were considered as potential confounders. These included opioids (ATC code: N02A*), antipsychotics (N05A*) excluding lithium (N05AN01), anxiolytics (N05B*) and hypnotics and sedatives (N05C*), antidepressants (N06A*), dopaminergic agents (N04B*), vasodilators (C01D*), antihypertensives (C02*), diuretics (C03*), beta blocking agents (C07*), calcium channel blockers (C08*), agents acting on the renin-angiotensin system (C09*), and alpha-adrenoreceptor antagonists (G04CA*) [26].

The use of anticholinesterases (N06DA*) was coded as a binary variable (yes/no) in each review period. Co-administration of anticholinergics and anticholinesterases, the most used pharmaceutical treatment for Alzheimer's disease should be avoided due to opposing actions [1, 27]. Anticholinesterases were considered as a potential confounder because: 1) older adults with dementia who are on anticholinesterases have increased risks of receiving anticholinergics than those not receiving anticholinesterases [28]; and 2) anticholinesterases are associated with decreased rates of falls [29].

### Outcome measures

The outcome measure was any falls (yes/no) in each 28-day review period after the one when anticholinergic load was measured. There was a total of 12 review periods as shown in Fig. 1. Falls were recorded in standardized electronic incident report forms and have been used in prior studies [26, 30]. As part of the larger program [16], the staff was present around the clock and made substantial efforts to record information on falls.

### Statistical analyses

Descriptive statistics were calculated. Associations between anticholinergic load on each scale in a review period and any falls in the next review period were determined using mixed-effect logistic models, taking into account 12 repeated observations within each resident and clustering by facility.

We conducted analyses for three models. These were unadjusted, then adjusted for age, sex, health conditions (considered as indications or contraindications for anticholinergics and related to falls [31], namely dementia, mood disorder, Parkinson’s disease, prior history of falls before RACF entry). For each resident, 12 repeated examinations of the association were coded in order as integers from zero to 11 and were controlled for. In the final models (fully adjusted), we additionally adjusted for the year in which residents entered the facility (coded as a continuous variable: integers 0 to 7 for each year between 2014 and 2021) and time dependent variables for each 28-day review period. Time dependent variables included resident type (“permanent” or “respite”, as many residents entered as “respite” and then changed to “permanent”), number of fall-risk-increasing medications that were not listed on the corresponding anticholinergic scale, whether residents were on any anticholinesterases, and number of medications that were none of the following: listed on the corresponding anticholinergic scale, fall-risk-increasing medications, and anticholinesterases. Length of exposure and outcome measures were controlled for, and included the number of days each resident stayed in the facility for the review periods: 1) when medication use was examined, and 2) when falls were examined. Natural logarithms were taken for both.

All analyses were conducted overall and separately for residents with and without dementia. Given the increased attention to medication use and falls in RACFs in recent years [32], a sensitivity analysis was conducted to examine associations between anticholinergic load and falls, for subsets of residents by the year when they entered the RACF. Results were presented as odds ratios (OR) with 95% confidence interval (CI), with *p* < 0.05 regarded as statistically significant. Stata MP 18 (StataCorp LP, College Station, TX) was used for analyses.

## Results

We excluded 1752 residents, who did not stay in a facility for at least one day in one or more review periods. The characteristics of excluded residents are presented in Tables S2 and S3. Among those who were excluded from the analyses, 494 (28.2%) had their initial resident type recorded as permanent, compared to 1179 (51.3%) among the study sample (Table S3).

**Fig. 2:**
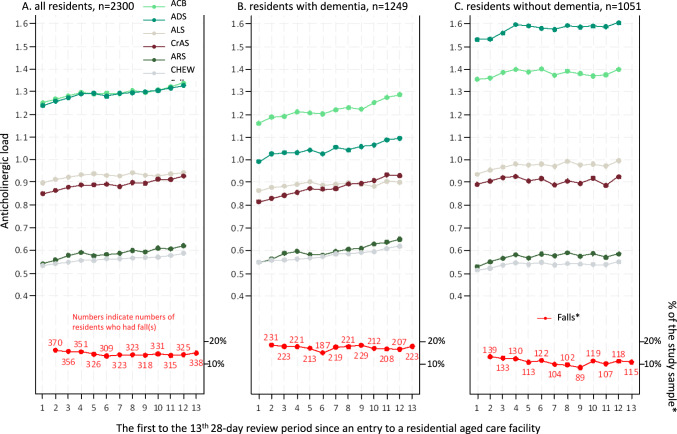
Anticholinergic load and percentages and numbers of residents who had fall(s). *ACB* Anticholinergic Cognitive Burden, *ADS* Anticholinergic Drug Scale, *ALS* Anticholinergic Loading Scale, *ARS* Anticholinergic Risk Scale, *CHEW* Chew’s list, *CrAS* Clinician-rated Anticholinergic Score. *y-axis is on the right

A total of 2300 residents and 27,600 resident-periods were included (Tables S2 to S4). There were steady increases in mean anticholinergic load from the first to the 12th review period, by all six measures (Fig. 2). Supplementary Table S5 shows administered anticholinergic medications. For each of the second to 13th review period, percentages of residents who experienced falls ranged from 13.4% (*n* = 309, the 6th review period) to 16.1% (*n* = 370, the 2nd review period) (Fig. 2).

**Fig. 3:**
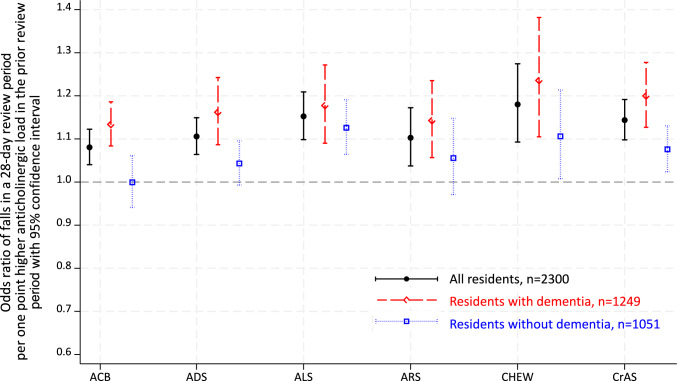
Odds ratios for per one-point higher anticholinergic load in the current review period and falls in the next review period. *ACB* Anticholinergic Cognitive Burden, *ADS* Anticholinergic Drug Scale, *ALS* Anticholinergic Loading Scale, *ARS* Anticholinergic Risk Scale, *CHEW* Chew’s list, *CrAS* Clinician-rated Anticholinergic Score

Results were similar in unadjusted to the final models (Tables S6 to S8). In the final models, a one-point increase in anticholinergic load (as measured using each of six scales, in any of the first to the 12th review period), was related to 8% to 18% higher risks of falls in the following review period (Fig. 3, Tables S6 to S8). ORs were 1.08 (95%CI 1.04, 1.12) (ACB), 1.11 (95%CI 1.06, 1.15) (ADS), 1.15 (95%CI 1.10, 1.21) (ALS), 1.10 (95%CI 1.04, 1.17) (ARS), 1.18 (95%CI 1.09, 1.27) (Chew’s list), and 1.14 (95%CI 1.10, 1.19) (CrAS). These associations remained statistically significant in the subgroup analysis for residents with dementia. For residents without dementia, no associations were found between anticholinergic load, as measured using the ACB, ADS, and ARS, and falls, whereas ORs were 1.13 (95%CI 1.06, 1.19) measured using ALS, 1.11 (95%CI 1.01, 1.21) using Chew’s list, and 1.08 (95%CI 1.02, 1.13) using CrAS.

In the sensitivity analysis, we observed that, when measured using ACB and ADS, mean anticholinergic load in most of the review periods were below 1.2 for residents who entered in year 2020 or 2021, compared to above 1.2 for residents who entered earlier than 2020 (Figure S1). Similar associations between anticholinergic load and falls were found among residents who entered the RACF between 2014 and 2021 (Table S9). Yet, in these subsets of the study sample, many of the associations attenuated to null.

## Discussion

In this longitudinal study, we found slight increases in residents’ anticholinergic load over time in the first year after entry to a RACF. A one-point higher anticholinergic load by any of six studied measures, in any of the first 12 review periods after admission, was associated with 8% to 18% higher risk of falls in the following 28 days. Residents with dementia were at 13% to 24% higher risk of falls per one-point increase in anticholinergic load, using each of the scales. In residents without dementia, risks were attenuated and anticholinergic load measured on the ACB, ADS, and ARS was not associated with falls.

Estimations of fall-risk in our study are not directly comparable to other published literature due to differences in study designs. A one-point higher anticholinergic load based on self-reported medication use at baseline was related to greater risks of hospital admission for a fall or fracture, using data from the UK Biobank [10], with hazard ratios of 1.11 for the ACB, 1.13 for the ADS, 1.10 for the ALS, 1.06 for the ARS, 1.09 for Chew’s list, and 1.09 for the CrAS [10]. A one-point higher anticholinergic load was related to higher risks of falls in home-dwelling older adults in Germany by some measures: 19% (ALS), 37% (ARS), and 14% (Chew’s list), but not others (ACB, ADS, or CrAS). In that study, the authors reviewed medication use at a single time point, and retrospectively reviewed self-reported history of falls in the previous 12 months [19]. In a population-based New Zealand study of those aged ≥ 65 years, a one-point higher daily anticholinergic load in 2011 was related to higher rates of hospitalizations for falls in 2012, incident rate ratios of 1.14 by the Chew’s list and 1.20 to 1.22 for the other five measures we studied [9]. Our ORs considered a shorter duration between the exposure and outcome, which might be more clinically relevant [17]. An earlier systematic review on anticholinergic load and falls in older adults not including the UK Biobank [10], German [19], and New Zealand studies [9], found that out of eight of their included studies, ACB and ARS were used by six and two studies, respectively [5]. All included studies showed a positive association between anticholinergic load and falls [5].

We observed potentially lower anticholinergic load measured using ACB and ADS among residents who entered in year 2020 and 2021, than those who entered earlier. This may be an improvement observed following a few policy implementations since 2018. For instance, a Royal Commission into Aged Care Quality and Safety was established in 2018 [33]. Their interim and final reports published in 2019 and 2021 drew significant attention to care and services provided to RACF residents [33]. During the Royal Commission, the Australian Government Department of Health and Aged Care introduced a National Aged Care Mandatory Quality Indicator Program in 2019, requiring RACF to monitor and report on quality indicators. Quality indicators of falls, polypharmacy, and antipsychotics were added to this program in 2021 [32]. Additionally, there were restrictions applied on the risperidone in the Australian Government’s Pharmaceutical Benefits Scheme. From January 2020, individuals with Alzheimer’s disease are only qualified for subsidized treatment of risperidone once every 12 months, for behavioral and psychological symptoms, and the use is restricted to ≤ 12 weeks [34]. However, the reductions in anticholinergic load in our study sample over time, if any, might be too subtle, as measurable steeper deterioration in clinical outcomes, e.g., cognitive function, would need a two-point increase in ACB score [35]. We did not observe dramatic changes in rates of residents who had fall(s) among those who entered between year 2014 and 2021. However, an earlier study from the same RACFs but including residents who resided for over 30 days between 2019 and 2021 found that compared to the pre-COVID period (March 2019 to February 2020), falls increased by 32% during the first lockdown in New South Wales, Australia (March to June 2020), but remained unchanged in the second lockdown (December 2020 and January 2021) [36]. In our study, we found the association between anticholinergic load and falls remained consistent across the eight-year study period irrespective of temporal changes in anticholinergic load and falls.

A strength of our study was the use of high-quality medication administration data for a large sample of residents. By using medication administration data rather than prescription data, our data analyses considered medication adherence and dynamic medication use. Additionally, this is the first study adopting prospective and routinely collected data on falls, including falls not requiring hospitalization. There are some limitations. First, residents in permanent care are usually those who require continuous support, lasting months or years. Respite care, by contrast, provides temporary support to residents’ usual caregivers. We excluded those who did not stay at the facility for at least one day during any review period, and our results may not be generalizable to residents in short-term care and those who died soon after RACF entry. Second, minimum recommended daily dose and actual daily dose of anticholinergic medications were not taken into consideration [9]. Load from low-dose use may have been overestimated, whereas load from high-dose use may have been underestimated. However, simplicity of anticholinergic scales makes them practical for clinical and research application. Finally, since in 96% of the resident periods, no or only one fall was recorded, we did not examine repeated falls in the same review period.

## Implications and conclusion

Given the high rates of falls in residential aged care, and the increased availability of electronic medication systems in aged care facilities, we recommend the inclusion of an anticholinergic scale be incorporated to automatically identify residents who may be at increased risk of falls due to the use of these medicines. The system automatically sums scores for each resident in each review period and triggers warnings when there is an increase in a resident’s anticholinergic load. The ALS is the only scale developed in Australia [22]. We showed that anticholinergic load measured on this scale had similar associations with falls in residents with dementia compared to in those without dementia, with good statistical precision (tight CIs).

For new RACF residents, in the first year following RACF entry, associations were found between anticholinergic load in a 28-day review period and falls in the following 28-day period, after controlling for fall-risk-increasing medications use. This finding suggests that reducing anticholinergic load may be a suitable strategy for fall risk assessment and reduction, especially in people with dementia. However, due to variations in guidelines, prescribing habits, and availability of medications between different countries and regions, the best choice of anticholinergic scale may differ between populations.

## Supplementary Information

Below is the link to the electronic supplementary material.

Supplementary file1 (DOCX 306 KB)

## Data Availability

The data for this study are not publicly available. All authors have full access to the data, but do not have permission from the residents or ethics approval to share the data.
