# Supplementary material for: Anticholinergic medication use and falls in Australian residential aged care: a retrospective multisite cohort study
Source: Aging Clin Exp Res. 2025 Aug 27;37(1):257. doi: 10.1007/s40520-025-03147-9 (PMC12390879; doi:10.1007/s40520-025-03147-9)
Supplement: Supplementary file 1 — Supplementary file1 (DOCX 306 KB) [file 40520_2025_3147_MOESM1_ESM.docx]

Supplementary

This supplementary file is to provide additional information for article: Anticholinergic medication use and falls in Australian residential aged care: a retrospective multisite study

Table of Contents

[Table S1 Six anticholinergic scales used in the study 2](#_Toc203246854)

[Table S2 Characteristics of the study sample compared to those excluded from the analyses and by dementia status: time invariant variables 3](#_Toc203246855)

[Table S3 Characteristics of the study sample in the first review period, compared to those excluded from the analyses 4](#_Toc203246856)

[Table S4 Characteristics of the study sample: time dependent variables 5](#_Toc203246857)

[Table S5 Administered anticholinergic medications 6](#_Toc203246858)

[Table S6 Associations between per one-point higher anticholinergic load in a review period and falls in the next review period (all residents) 11](#_Toc203246859)

[Table S7 Associations between per one-point higher anticholinergic load in a review period and falls in the next review period (residents with dementia) 12](#_Toc203246860)

[Table S8 Associations between per one-point higher anticholinergic load in a review period and falls in the next review period (residents without dementia) 13](#_Toc203246861)

[Table S9 Associations between per one-point higher anticholinergic load in a review period and falls in the next review period by the year when they entered the facility 14](#_Toc203246862)

[Figure S1 Anticholinergic load and percentages and numbers of residents who had falls, overall and by the year when they entered the facility 16](#_Toc203246863)

# Table S1 Six anticholinergic scales used in the study

| Scale, country, ratings, year, first author | Details on studies where the scale was developed | |
| --- | --- | --- |
|  | Study sample | Key findings |
| Anticholinergic Loading Scale Australia (ALS)  0, 1, 2, 3  2011  Sittironnarit | The Australian Imaging, Biomarkers and Lifestyle study of ageing: cohort of individuals aged ≥60 years. Baseline data were used. | Higher anticholinergic load was cross-sectionally associated with lower psychomotor speed and executive function, but not on other areas of cognition in cognitively healthy older adults. It was not associated with cognition in participants with mild cognitive impairment or Alzheimer’s disease. |
| Anticholinergic Cognitive Burden (ACB)  USA  1, 2, 3  2008  Boustani | A Medline search identified studies evaluating anticholinergic use and relationships between anticholinergics and cognitive impairment. | In this review, the authors suggested to consider alternative medications with ACB <3 and reduce a total ACB score to <3. |
| Anticholinergic Drug Scale (ADS)  USA  0, 1, 2, 3  2006  Carnahan | Residents in rural long-term care facilities (n=201, aged 64 to 102 years). | Higher ADS load was associated with higher serum anticholinergic activity. A 1:2:3 proportion for the scores on the ADS is reasonable. |
| Anticholinergic Risk Scale (ARS)  USA  1, 2, 3  2008  Rudolph | A retrospective cohort of 132 participants seen consecutively in geriatric evaluation and management clinics at the Veterans Affairs Boston Healthcare System and a prospective cohort of 117 male participants from a primary care cohort, all aged ≥65 years. | Higher ARS load was associated with greater rates of having central (any of falls, dizziness, and confusion) and peripheral anticholinergic adverse effects (any of dry mouth, dry eyes, and constipation). |
| Chew’s list  USA  0, 0/+, +, ++, +++  2008  Chew | In vitro (a radioreceptor assay) anticholinergic activity of 107 prescription and over-the-counter medications at various therapeutic concentrations representative of dose ranges commonly used in elderly people. | Number of medications demonstrated dose-dependent anticholinergic activity: 22 (+, ++, +++); number of medications demonstrated anticholinergic activity at the highest concentrations: 17 (0/+). The 0/+ ones were coded as 0, to be consistent with an earlier study, doi: 10.1370/afm.2501. |
| Clinician-rated Anticholinergic Score (CrAS)  Canada  0, 1, 2, 3  2008  Han | Connecticut Veterans Longitudinal Cohort: 767 veterans aged ≥65 years recruited at a Department of Veterans Affairs primary care clinic. | Higher cumulative exposure to anticholinergic medications over one year was associated with lower memory and executive function, adjusting for baseline memory and executive function and other covariates. |

# Table S2 Characteristics of the study sample compared to those excluded from the analyses and by dementia status: time invariant variables

| **Characteristics** | **Excluded from the analyses** | **Study sample, included in the analyses** | | |
| --- | --- | --- | --- | --- |
|  | ***n* = 1752** | **Total *n* = 2300** | **Residents with dementia n = 1249** | **Residents without dementia n = 1051** |
| Age in years, mean ± SD | 85.4 ± 7.5 | 85.8 ± 7.1 | 85.0 ± 6.8 | 86.6 ± 7.4 |
| Age groups, n (%) |  |  |  |  |
| 65-74 years | 156 (8.9) | 192 (8.3) | 108 (8.6) | 84 (8.0) |
| 75-84 years | 552 (31.5) | 714 (31.0) | 438 (35.1) | 276 (26.3) |
| 85-94 years | 874 (49.9) | 1178 (51.2) | 613 (49.1) | 565 (53.8) |
| ≥95 years | 170 (9.7) | 216 (9.4) | 90 (7.2) | 126 (12.0) |
| Females, n (%) | 1020 (58.2) | 1556 (67.7) | 841 (67.3) | 715 (68.0) |
| Health conditions recorded at entries to a residential aged care facility, n (%) | | | |  |
| Dementia | 687 (39.2) | 1249 (54.3) | 1249 (100.0) | 0 (0) |
| Mood disorder^a^ | 691 (39.4) | 1252 (54.4) | 663 (53.1) | 589 (56.0) |
| Parkinson’s disease | 142 (8.1) | 178 (7.7) | 97 (7.8) | 81 (7.7) |
| History of falls | 778 (44.4) | 1239 (53.9) | 685 (54.8) | 554 (52.7) |
| Year when residents entered the facility, n (%)^b^ | | |  |  |
| 2014 | 136 (7.8) | 138 (6.0) | 95 (7.6) | 43 (4.1) |
| 2015 | 228 (13.0) | 290 (12.6) | 179 (14.3) | 111 (10.6) |
| 2016 | 238 (13.6) | 236 (10.3) | 132 (10.6) | 104 (9.9) |
| 2017 | 251 (14.3) | 353 (15.3) | 205 (16.4) | 148 (14.1) |
| 2018 | 263 (15.0) | 313 (13.6) | 156 (12.5) | 157 (14.9) |
| 2019 | 268 (15.3) | 369 (16.0) | 184 (14.7) | 185 (17.6) |
| 2020 | 180 (10.3) | 289 (12.6) | 146 (11.7) | 143 (13.6) |
| 2021 | 188 (10.7) | 312 (13.6) | 152 (12.2) | 160 (15.2) |

SD denotes standard deviation. Numbers are mean ± SD for continuous variables with normal distribution, or n (%) for categorical variables.

^a^Mood disorder included depression, anxiety, bipolar disorder, post-traumatic stress disorder, or mood disorder that was not specified.

^b^Coded as integers 0 to 7 and used as a continuous variable in mixed-effect logistic regression models.

# Table S3 Characteristics of the study sample in the first review period compared to those excluded from the analyses

| **Characteristics** | | **Excluded *n* = 1752** | **Included *n* = 2300** |
| --- | --- | --- | --- |
| Anticholinergic load, median (Q1, Q3) | ACB | 1 (0, 2) | 1 (0, 2) |
|  | ADS | 1 (0, 2) | 1 (0, 2) |
|  | ALS | 1 (0, 1) | 1 (0, 1) |
|  | ARS | 0 (0, 1) | 0 (0, 1) |
|  | CHEW | 0 (0, 1) | 0 (0, 1) |
|  | CrAS | 1 (0, 1) | 0 (0, 1) |
| Daily number of Fall-Risk-Increasing medications not listed in the corresponding scale, median (Q1, Q3) | ACB | 1 (0, 2) | 1 (0, 2) |
|  | ADS | 1 (0, 2) | 1 (0, 2) |
|  | ALS | 1 (1, 2) | 1 (1, 2) |
|  | ARS | 1 (1, 2) | 1 (1, 2) |
|  | CHEW | 1 (1, 2) | 1 (1, 2) |
|  | CrAS | 1 (0, 2) | 1 (0, 2) |
| Use of anticholinesterases, n (%) | | 69 (3.9) | 163 (7.1) |
| Permanent (as opposed to respite) care, n (%) | | 494 (28.2) | 1179 (51.3) |
| Daily number of other medications that were not anticholinesterases, not considered as Fall-Risk-Increasing medications, and not listed in the corresponding scale, mean ± SD | ACB | 4.8 ± 2.5 | 4.8 ± 2.5 |
|  | ADS | 4.7 ± 2.5 | 4.7 ± 2.4 |
|  | ALS | 4.8 ± 2.5 | 4.8 ± 2.5 |
|  | ARS | 4.9 ± 2.6 | 4.9 ± 2.5 |
|  | CHEW | 5.0 ± 2.6 | 4.9 ± 2.6 |
|  | CrAS | 4.9 ± 2.6 | 4.9 ± 2.6 |
| Length of the first review period (in days), mean ± SD, median (Q1, Q3) | | 23.6 ± 6.1  26 (22, 28) | 25.6 ± 4.1  27 (25, 28) |

ACB denotes Anticholinergic Cognitive Burden, ADS Anticholinergic Drug Scale, ALS Anticholinergic Loading Scale, ARS Anticholinergic Risk Scale, CHEW Chew’s list, CrAS Clinician-rated Anticholinergic Score, Q1 the first quartile, Q3 the third quartile, and SD standard deviation.

Numbers are mean ± SD for continuous variables with normal distribution, median (Q1 lower quartile, Q3 upper quartile) for continuous variables with skewed distribution, or n (%) for categorical variables. Both mean ± SD and median (Q1, Q3) were reported for the variable of “length of the first review period”, with a skewed distribution.

# Table S4 Characteristics of the study sample: time dependent variables

| **Characteristics** | | **Total resident-review-period** | | |
| --- | --- | --- | --- | --- |
|  |  | **all** | **with dementia** | **without dementia** |
|  |  | **27,600** | **14988** | **12612** |
| Anticholinergic load, median (Q1, Q3) | ACB | 1 (0, 2) | 1 (0, 2) | 1 (0, 2) |
|  | ADS | 1 (0, 2) | 1 (0, 2) | 1 (0, 2) |
|  | ALS | 1 (0, 1) | 1 (0, 1) | 1 (0, 2) |
|  | ARS | 0 (0, 1) | 0 (0, 1) | 0 (0, 1) |
|  | CHEW | 0 (0, 1) | 0 (0, 1) | 0 (0, 1) |
|  | CrAS | 0 (0, 1) | 1 (0, 1) | 0 (0, 1) |
| Daily number of Fall-Risk-Increasing medications not listed in the corresponding scale, median (Q1, Q3) | ACB | 1.0 (0.3, 2.0) | 1.0 (0.0, 1.9) | 1.0 (0.7, 2.0) |
|  | ADS | 1.0 (0.5, 2.0) | 1.0 (0.4, 2.0) | 1.0 (0.7, 2.0) |
|  | ALS | 1.0 (0.7, 2.0) | 1.0 (0.3, 2.0) | 1.6 (0.9, 2.5) |
|  | ARS | 1.3 (0.8, 2.3) | 1.0 (0.5, 2.0) | 1.9 (1.0, 2.8) |
|  | CHEW | 1.1 (0.6, 2.0) | 1.0 (0.2, 2.0) | 1.7 (0.9, 2.6) |
|  | CrAS | 1.0 (0.5, 2.0) | 1.0 (0.1, 1.9) | 1.3 (0.8, 2.1) |
| Use of anticholinesterases, n (%) | | 2244 (8.1) | 2244 (15.0) | 0 (0) |
| Permanent (as opposed to respite) care, n (%) | | 25523 (92.5) | 13900 (92.7) | 11623 (92.2) |
| Daily number of other medications that were not anticholinesterases, not considered as Fall-Risk-Increasing medications, and not listed in the corresponding scale, mean ± SD | ACB | 5.3 ± 2.7 | 4.9 ± 2.6 | 5.8 ± 2.8 |
|  | ADS | 5.2 ± 2.7 | 4.8 ± 2.5 | 5.7 ± 2.8 |
|  | ALS | 5.3 ± 2.7 | 4.9 ± 2.5 | 5.8 ± 2.8 |
|  | ARS | 5.5 ± 2.8 | 5.0 ± 2.6 | 6.0 ± 2.9 |
|  | CHEW | 5.5 ± 2.8 | 5.1 ± 2.6 | 6.0 ± 2.9 |
|  | CrAS | 5.5 ± 2.8 | 5.1 ± 2.6 | 6.0 ± 2.9 |
| Length of review period (in days) when measurements of medication use were examined, mean ± SD, median (Q1, Q3) | | 27.4 ± 2.5  28 (28, 28) | 27.5 ± 2.3  28 (28, 28) | 27.3 ± 2.7  28 (28, 28) |
| Length of review period (in days) when falls were examined**^a^**, mean ± SD, median (Q1, Q3) | | 27.5 ± 2.3  28 (28, 28) | 27.6 ± 2.1  28 (28, 28) | 27.4 ± 2.5  28 (28, 28) |

ACB denotes Anticholinergic Cognitive Burden, ADS Anticholinergic Drug Scale, ALS Anticholinergic Loading Scale, ARS Anticholinergic Risk Scale, CHEW Chew’s list, CrAS Clinician-rated Anticholinergic Score, Q1 the first quartile, Q3 the third quartile, and SD standard deviation.

Numbers are mean ± SD for continuous variables with normal distribution, median (Q1 lower quartile, Q3 upper quartile) for continuous variables with skewed distribution, or n (%) for categorical variables. Both mean ± SD and median (Q1, Q3) were reported for the variable of “length of the first review period”, with a skewed distribution.

^a^Each resident had 12 repeated examinations (Figure 1). This variable, “length of review period (in days) when falls were examined”, was referred to the second to the 13^th^ 28-day review periods. All other variables listed in this table were referred to the first to the 12^th^ 28-day review periods.

# Table S5 Administered anticholinergic medications

| ATC code, generic name | Residents, *n* (%) | | | | Resident-review-periods, *n* (%) | | | Scores by scale | | | | | |
| --- | --- | --- | --- | --- | --- | --- | --- | --- | --- | --- | --- | --- | --- |
|  | All residents | Dementia | No dementia | All residents | | Dementia | No dementia | ACB | ADS | ALS | ARS | CHEW | CRA |
|  | *n*=2330 | *n*=1249 | *n*=1051 | *n*=27600 | | *n*=14988 | *n*=12612 |  |  |  |  |  |  |
| A02BA02 ranitidine | 57 (2.4) | 24 (1.9) | 33 (3.1) | 411 (1.5) | | 178 (1.2) | 233 (1.8) | 1 | 2 | 1 | 1 | 1 | 2 |
| A02BA03 famotidine | 4 (0.2) | 1 (0.1) | 3 (0.3) | 42 (0.2) | | 12 (0.1) | 30 (0.2) |  | 1 |  |  |  |  |
| A02BA04 nizatidine | 23 (1.0) | 5 (0.4) | 18 (1.7) | 166 (0.6) | | 31 (0.2) | 135 (1.1) |  | 1 |  |  |  |  |
| A03FA01 metoclopramide | 152 (6.5) | 57 (4.6) | 95 (9.0) | 454 (1.6) | | 161 (1.1) | 293 (2.3) |  |  | 1 | 1 |  | 3 |
| A03FA03 domperidone | 57 (2.4) | 16 (1.3) | 41 (3.9) | 410 (1.5) | | 120 (0.8) | 290 (2.3) |  |  | 1 |  |  |  |
| A06AB02 bisacodyl | 36 (1.5) | 21 (1.7) | 15 (1.4) | 197 (0.7) | | 136 (0.9) | 61 (0.5) |  |  | 1 |  |  |  |
| A07DA03 loperamide | 50 (2.1) | 17 (1.4) | 33 (3.1) | 216 (0.8) | | 54 (0.4) | 162 (1.3) | 1 | 1 | 1 | 2 |  | 1 |
| A10BA02 metformin | 230 (9.9) | 130 (10.4) | 100 (9.5) | 2307 (8.4) | | 1308 (8.7) | 999 (7.9) |  |  | 1 |  |  |  |
| B01AA03 warfarin | 175 (7.5) | 70 (5.6) | 105 (10.0) | 1814 (6.6) | | 741 (4.9) | 1073 (8.5) | 1 | 1 |  |  |  |  |
| B01AC07 dipyridamole | 4 (0.2) | 1 (0.1) | 3 (0.3) | 48 (0.2) | | 12 (0.1) | 36 (0.3) | 1 | 1 |  |  |  |  |
| C01AA05 digoxin | 207 (8.9) | 100 (8.0) | 107 (10.2) | 2259 (8.2) | | 1088 (7.3) | 1171 (9.3) | 1 | 1 | 1 |  |  |  |
| C01DA14 isosorbide mononitrate | 108 (4.6) | 45 (3.6) | 63 (6.0) | 1157 (4.2) | | 486 (3.2) | 671 (5.3) | 1 | 1 |  |  |  |  |
| C02DB02 hydralazine | 21 (0.9) | 5 (0.4) | 16 (1.5) | 173 (0.6) | | 45 (0.3) | 128 (1.0) | 1 | 1 |  |  |  |  |
| C03CA01 furosemide | 762 (32.7) | 307 (24.6) | 455 (43.3) | 6991 (25.3) | | 2667 (17.8) | 4324 (34.3) | 1 | 1 |  |  |  |  |
| C07AB02 metoprolol | 354 (15.2) | 180 (14.4) | 174 (16.6) | 3845 (13.9) | | 2024 (13.5) | 1821 (14.4) | 1 |  |  |  |  | 1 |
| C07AB03 atenolol | 116 (5.0) | 62 (5.0) | 54 (5.1) | 1313 (4.8) | | 705 (4.7) | 608 (4.8) | 1 |  |  |  |  | 1 |
| C08CA05 nifedipine | 17 (0.7) | 7 (0.6) | 10 (1.0) | 164 (0.6) | | 62 (0.4) | 102 (0.8) | 1 | 1 |  |  |  |  |
| C08DB01 diltiazem | 61 (2.6) | 28 (2.2) | 33 (3.1) | 627 (2.3) | | 302 (2.0) | 325 (2.6) |  | 1 |  |  |  |  |
| C09AA10 trandolapril | 7 (0.3) | 4 (0.3) | 3 (0.3) | 76 (0.3) | | 48 (0.3) | 28 (0.2) |  |  |  |  |  | 1 |
| G04BD04 oxybutynin | 69 (3.0) | 27 (2.2) | 42 (4.0) | 595 (2.2) | | 204 (1.4) | 391 (3.1) | 3 | 3 | 2 | 3 | 2 |  |
| G04BD07 tolterodine | 2 (0.1) | 1 (0.1) | 1 (0.1) | 7 (0.03) | | 5 (0.03) | 2 (0.02) | 3 | 3 | 3 | 2 | 3 | 3 |
| G04BD10 darifenacin | 2 (0.1) | 1 (0.1) | 1 (0.1) | 18 (0.1) | | 12 (0.1) | 6 (0.05) | 3 | 3 |  |  |  |  |
| H02AB02 dexamethasone | 12 (0.5) | 4 (0.3) | 8 (0.8) | 58 (0.2) | | 9 (0.1) | 49 (0.4) |  | 1 |  |  |  |  |
| H02AB04 H02BX01 methylprednisolone | 1 (0.04) | 1 (0.1) | 0 (0) | 1 (0.004) | | 1 (0.01) | 0 (0) |  | 1 |  |  |  |  |
| H02AB06 prednisolone | 107 (4.6) | 43 (3.4) | 64 (6.1) | 628 (2.3) | | 286 (1.9) | 342 (2.7) |  | 1 |  |  |  |  |
| H02AB07 prednisone | 192 (8.2) | 68 (5.4) | 124 (11.8) | 1140 (4.1) | | 400 (2.7) | 740 (5.9) | 1 | 1 |  |  |  |  |
| H02AB09 hydrocortisone | 2 (0.1) | 1 (0.1) | 1 (0.1) | 13 (0.05) | | 12 (0.1) | 1 (0.01) | 1 | 1 |  |  |  |  |
| H02AB10 cortisone | 3 (0.1) | 0 (0) | 3 (0.3) | 36 (0.1) | | 0 (0) | 36 (0.3) |  | 1 |  |  |  |  |
| J01FF01 clindamycin | 101 (4.3) | 40 (3.2) | 61 (5.8) | 190 (0.7) | | 89 (0.6) | 101 (0.8) |  | 1 |  |  |  |  |
| J01GB03 gentamicin | 1 (0.04) | 1 (0.1) | 0 (0) | 1 (0.004) | | 1 (0.01) | 0 (0) |  | 1 |  |  |  |  |
| J01XA01 vancomycin | 1 (0.04) | 1 (0.1) | 0 (0) | 2 (0.007) | | 2 (0.01) | 0 (0) |  | 1 |  |  |  |  |
| L04AD01 ciclosporin | 1 (0.04) | 0 (0) | 1 (0.1) | 12 (0.04) | | 0 (0) | 12 (0.1) |  | 1 |  |  |  |  |
| L04AX01 azathioprine | 3 (0.1) | 2 (0.2) | 1 (0.1) | 32 (0.1) | | 20 (0.1) | 12 (0.1) |  | 1 |  |  |  |  |
| L04AX03 methotrexate | 26 (1.1) | 10 (0.8) | 16 (1.5) | 227 (0.8) | | 90 (0.6) | 137 (1.1) |  |  | 1 |  |  |  |
| M01AH01 celecoxib | 43 (1.8) | 21 (1.7) | 22 (2.1) | 284 (1.0) | | 134 (0.9) | 150 (1.2) |  |  | 1 |  |  |  |
| M03BX01 baclofen | 12 (0.5) | 4 (0.3) | 8 (0.8) | 119 (0.4) | | 37 (0.2) | 82 (0.7) |  |  |  | 2 |  | 2 |
| M04AC01 colchicine | 40 (1.7) | 19 (1.5) | 21 (2.0) | 216 (0.8) | | 104 (0.7) | 112 (0.9) | 1 |  |  |  |  |  |
| N02AA01 morphine | 65 (2.8) | 27 (2.2) | 38 (3.6) | 294 (1.1) | | 92 (0.6) | 202 (1.6) | 1 | 1 |  |  |  | 1 |
| N02AA05 oxycodone | 481 (20.6) | 220 (17.6) | 261 (24.8) | 2350 (8.5) | | 965 (6.4) | 1385 (11.0) |  | 1 | 1 |  |  | 1 |
| N02AA59 codeine | 1 (0.04) | 0 (0) | 1 (0.1) | 4 (0.01) | | 0 (0) | 4 (0.03) | 1 | 1 | 1 |  |  | 1 |
| N02AB03 fentanyl | 44 (1.9) | 19 (1.5) | 25 (2.4) | 326 (1.2) | | 105 (0.7) | 221 (1.8) | 1 | 1 |  |  |  |  |
| N02AJ06 codeine | 85 (3.6) | 35 (2.8) | 50 (4.8) | 428 (1.6) | | 145 (1.0) | 283 (2.2) | 2 | 1 | 1 |  |  | 1 |
| N02AX02 tramadol | 46 (2.0) | 18 (1.4) | 28 (2.7) | 300 (1.1) | | 126 (0.8) | 174 (1.4) |  | 1 | 2 |  |  | 2 |
| N02CC01 sumatriptan | 1 (0.04) | 0 (0) | 1 (0.1) | 8 (0.03) | | 0 (0) | 8 (0.1) |  |  | 1 |  |  |  |
| N03AE01 clonazepam | 42 (1.8) | 27 (2.2) | 15 (1.4) | 327 (1.2) | | 194 (1.3) | 133 (1.1) |  | 1 | 1 |  |  |  |
| N03AF01 carbamazepine | 22 (0.9) | 13 (1.0) | 9 (0.9) | 243 (0.9) | | 146 (1.0) | 97 (0.8) | 2 | 2 |  |  |  | 1 |
| N03AG01 valproic acid | 124 (5.3) | 89 (7.1) | 35 (3.3) | 1133 (4.1) | | 777 (5.2) | 356 (2.8) |  | 1 |  |  |  |  |
| N04AA01 trihexyphenidyl | 1 (0.04) | 0 (0) | 1 (0.1) | 6 (0.02) | | 0 (0) | 6 (0.05) | 3 | 3 |  |  |  | 3 |
| N04AC01 benzatropine | 3 (0.1) | 2 (0.2) | 1 (0.1) | 36 (0.1) | | 24 (0.2) | 12 (0.1) | 3 | 3 |  | 3 |  |  |
| N04BB01 amantadine | 2 (0.1) | 2 (0.2) | 0 (0) | 14 (0.1) | | 14 (0.1) |  | 2 | 1 |  | 2 |  |  |
| N04BC05 pramipexole | 41 (1.8) | 15 (1.2) | 26 (2.5) | 442 (1.6) | | 159 (1.1) | 283 (2.2) |  |  |  | 1 |  |  |
| N04BD01 selegiline | 3 (0.1) | 3 (0.2) | 0 (0) | 29 (0.1) | | 29 (0.2) | 0 (0) |  |  |  | 1 |  |  |
| N04BX02 entacapone | 1 (0.04) | 1 (0.1) | 0 (0) | 12 (0.04) | | 12 (0.1) | 0 (0) |  |  |  | 1 |  |  |
| N05AA01 chlorpromazine | 5 (0.2) | 1 (0.1) | 4 (0.4) | 47 (0.2) | | 12 (0.1) | 35 (0.3) | 3 | 3 |  | 3 | 2 | 3 |
| N05AB04 prochlorperazine | 66 (2.8) | 23 (1.8) | 43 (4.1) | 232 (0.8) | | 105 (0.7) | 127 (1.0) |  | 1 | 2 | 2 |  | 2 |
| N05AD01 haloperidol | 48 (2.1) | 40 (3.2) | 8 (0.8) | 300 (1.1) | | 260 (1.7) | 40 (0.3) | 1 |  | 2 | 1 |  |  |
| N05AH02 clozapine | 1 (0.04) | 1 (0.1) | 0 (0) | 12 (0.04) | | 12 (0.1) | 0 (0) | 3 | 3 |  | 2 | 3 |  |
| N05AH03 olanzapine | 77 (3.3) | 56 (4.5) | 21 (2.0) | 714 (2.6) | | 522 (3.5) | 192 (1.5) | 3 | 1 |  | 2 | 2 | 1 |
| N05AH04 quetiapine | 157 (6.7) | 127 (10.2) | 30 (2.9) | 1223 (4.4) | | 960 (6.4) | 263 (2.1) | 3 |  |  | 1 | 1 | 2 |
| N05AN01 lithium | 5 (0.2) | 2 (0.2) | 3 (0.3) | 59 (0.2) | | 23 (0.2) | 36 (0.3) |  |  | 1 |  | 1 |  |
| N05AX08 risperidone | 203 (8.7) | 183 (14.7) | 20 (1.9) | 1525 (5.5) | | 1365 (9.1) | 160 (1.3) | 1 |  | 1 | 1 |  | 1 |
| N05BA01 diazepam | 78 (3.3) | 33 (2.6) | 45 (4.3) | 465 (1.7) | | 192 (1.3) | 273 (2.2) | 1 | 1 | 1 |  |  | 1 |
| N05BA04 oxazepam | 30 (1.3) | 14 (1.1) | 16 (1.5) | 286 (1.0) | | 106 (0.7) | 180 (1.4) |  | 1 | 1 |  |  |  |
| N05BA06 lorazepam | 71 (3.0) | 53 (4.2) | 18 (1.7) | 481 (1.7) | | 334 (2.2) | 147 (1.2) |  | 1 |  |  |  |  |
| N05BA12 alprazolam | 9 (0.4) | 4 (0.3) | 5 (0.5) | 74 (0.3) | | 23 (0.2) | 51 (0.4) | 1 | 1 | 1 |  |  | 1 |
| N05CD07 temazepam | 277 (11.9) | 131 (10.5) | 146 (13.9) | 2074 (7.5) | | 966 (6.4) | 1108 (8.8) |  | 1 | 1 |  | 1 |  |
| N05CD08 midazolam | 16 (0.7) | 10 (0.8) | 6 (0.6) | 22 (0.1) | | 14 (0.1) | 8 (0.1) |  | 1 |  |  |  |  |
| N06AA09 amitriptyline | 81 (3.5) | 41 (3.3) | 40 (3.8) | 721 (2.6) | | 347 (2.3) | 374 (3.0) | 3 | 3 | 3 | 3 | 3 | 3 |
| N06AA10 nortriptyline | 10 (0.4) | 2 (0.2) | 8 (0.8) | 94 (0.3) | | 24 (0.2) | 70 (0.6) | 3 | 3 |  | 2 | 2 | 3 |
| N06AA12 doxepin | 11 (0.5) | 6 (0.5) | 5 (0.5) | 96 (0.3) | | 42 (0.3) | 54 (0.4) | 3 | 3 | 3 |  | 3 | 3 |
| N06AA16 dosulepin | 12 (0.5) | 8 (0.6) | 4 (0.4) | 131 (0.5) | | 83 (0.6) | 48 (0.4) |  |  | 2 |  |  |  |
| N06AB03 fluoxetine | 29 (1.2) | 14 (1.1) | 15 (1.4) | 294 (1.1) | | 126 (0.8) | 168 (1.3) |  | 1 | 1 |  | 1 | 1 |
| N06AB04 citalopram | 174 (7.5) | 123 (9.8) | 51 (4.9) | 1633 (5.9) | | 1131 (7.5) | 502 (4.0) |  |  | 1 |  | 1 |  |
| N06AB05 paroxetine | 15 (0.6) | 10 (0.8) | 5 (0.5) | 156 (0.6) | | 114 (0.8) | 42 (0.3) | 3 | 1 | 2 | 1 | 2 | 2 |
| N06AB06 sertraline | 147 (6.3) | 88 (7.0) | 59 (5.6) | 1371 (5.0) | | 817 (5.5) | 554 (4.4) |  | 1 |  |  |  | 1 |
| N06AB08 fluvoxamine | 2 (0.1) | 1 (0.1) | 1 (0.1) | 24 (0.1) | | 12 (0.1) | 12 (0.1) | 1 | 1 | 1 |  |  |  |
| N06AB10 escitalopram | 123 (5.3) | 75 (6.0) | 48 (4.6) | 1208 (4.4) | | 730 (4.9) | 478 (3.8) |  |  | 1 |  | 1 |  |
| N06AX11 mirtazapine | 310 (13.3) | 176 (14.1) | 134 (12.7) | 2865 (10.4) | | 1612 (10.8) | 1253 (9.9) |  |  |  | 1 | 1 |  |
| N06AX16 venlafaxine | 88 (3.8) | 46 (3.7) | 42 (4.0) | 875 (3.2) | | 459 (3.1) | 416 (3.3) |  |  | 1 |  |  | 1 |
| N07BC02 methadone | 3 (0.1) | 1 (0.1) | 2 (0.2) | 28 (0.1) | | 11 (0.1) | 17 (0.1) |  |  |  |  |  | 2 |
| R01BA02 pseudoephedrine | 1 (0.04) | 1 (0.1) | 0 (0) | 1 (0.004) | | 1 (0.01) | 0 (0) |  |  | 2 | 2 |  |  |
| R03AK06 salmeterol and fluticasone | 179 (7.7) | 65 (5.2) | 114 (10.8) | 1819 (6.6) | | 693 (4.6) | 1126 (8.9) |  | 1 |  |  |  |  |
| R03DA04 theophylline | 2 (0.1) | 2 (0.2) | 0 (0) | 23 (0.1) | | 23 (0.2) | 0 (0) | 1 | 1 | 2 |  |  |  |
| R05CA03 guaifenesin | 1 (0.04) |  | 1 (0.1) | 2 (0.01) | | 0 (0) | 2 (0.02) |  |  |  |  |  | 1 |
| R05DA04 codeine | 12 (0.5) | 8 (0.6) | 4 (0.4) | 44 (0.2) | | 30 (0.2) | 14 (0.1) | 1 | 1 | 1 |  |  | 1 |
| R05DA09 dextromethorphan | 1 (0.04) | 1 (0.1) | 0 (0) | 1 (0.004) | | 1 (0.01) | 0 (0) |  |  |  |  |  | 1 |
| R06AA02 diphenhydramine | 2 (0.1) | 1 (0.1) | 1 (0.1) | 4 (0.01) | | 2 (0.01) | 2 (0.02) | 3 | 3 |  | 3 | 2 | 3 |
| R06AB02 dexchlorpheniramine | 2 (0.1) | 1 (0.1) | 1 (0.1) | 2 (0.007) | | 1 (0.01) | 1 (0.01) |  |  | 3 |  |  |  |
| R06AD02 promethazine | 20 (0.9) | 9 (0.7) | 11 (1.0) | 110 (0.4) | | 57 (0.4) | 53 (0.4) | 3 | 3 |  | 3 |  |  |
| R06AE07 cetirizine | 35 (1.5) | 19 (1.5) | 16 (1.5) | 178 (0.6) | | 108 (0.7) | 70 (0.6) |  |  | 2 | 2 |  | 2 |
| R06AX13 loratadine | 122 (5.2) | 57 (4.6) | 65 (6.2) | 542 (2.0) | | 219 (1.5) | 323 (2.6) |  |  | 1 | 2 |  | 1 |
| R06AX26 fexofenadine | 46 (2.0) | 19 (1.5) | 27 (2.6) | 167 (0.6) | | 77 (0.5) | 90 (0.7) |  |  | 2 |  |  | 2 |
| S01GX08 ketotifen | 5 (0.2) | 1 (0.1) | 4 (0.4) | 7 (0.03) | | 1 (0.01) | 6 (0.05) |  | 1 |  |  |  |  |

ACB denotes Anticholinergic Cognitive Burden, ADS Anticholinergic Drug Scale, ALS Anticholinergic Loading Scale, ARS Anticholinergic Risk Scale, CHEW Chew’s list, and CrAS Clinician-rated Anticholinergic Score.

# Table S6 Associations between per one-point higher anticholinergic load in a review period and falls in the next review period (all residents)

| Scale | Unadjusted | | Adjusted for age, sex, health condition, and 12 repeated examinations of the association were coded in order as integers from zero to 11 | | Final model, additionally adjusted for the year in which residents entered the facility and time dependent variables | |
| --- | --- | --- | --- | --- | --- | --- |
|  | Odds ratio (95% CI) | p value | Odds ratio (95% CI) | p value | Odds ratio (95% CI) | p value |
| ACB | 1.07 (1.03, 1.12) | 0.002 | 1.08 (1.04, 1.13) | <0.0001 | 1.08 (1.04, 1.12) | <0.0001 |
| ADS | 1.07 (1.02, 1.13) | 0.005 | 1.11 (1.07, 1.15) | <0.0001 | 1.11 (1.06, 1.15) | <0.0001 |
| ALS | 1.14 (1.08, 1.21) | <0.0001 | 1.16 (1.10, 1.22) | <0.0001 | 1.15 (1.10, 1.21) | <0.0001 |
| ARS | 1.10 (1.04, 1.17) | 0.002 | 1.10 (1.04, 1.17) | 0.001 | 1.10 (1.04, 1.17) | 0.002 |
| CHEW | 1.18 (1.09, 1.28) | <0.0001 | 1.18 (1.10, 1.28) | <0.0001 | 1.18 (1.09, 1.27) | <0.0001 |
| CrAS | 1.13 (1.09, 1.18) | <0.0001 | 1.15 (1.10, 1.20) | <0.0001 | 1.14 (1.10, 1.19) | <0.0001 |

ACB denotes Anticholinergic Cognitive Burden, ADS Anticholinergic Drug Scale, ALS Anticholinergic Loading Scale, ARS Anticholinergic Risk Scale, CHEW Chew’s list, CrAS Clinician-rated Anticholinergic Score, and CI confidence interval.

# Table S7 Associations between per one-point higher anticholinergic load in a review period and falls in the next review period (residents with dementia)

| Scale | Unadjusted | | Adjusted for age, sex, health condition, and 12 repeated examinations of the association were coded in order as integers from zero to 11 | | Final model, additionally adjusted for the year in which residents entered the facility and time dependent variables | |
| --- | --- | --- | --- | --- | --- | --- |
|  | Odds ratio (95% CI) | p value | Odds ratio (95% CI) | p value | Odds ratio (95% CI) | p value |
| ACB | 1.14 (1.08, 1.20) | <0.0001 | 1.14 (1.09, 1.19) | <0.0001 | 1.13 (1.08, 1.19) | <0.0001 |
| ADS | 1.18 (1.10, 1.27) | <0.0001 | 1.17 (1.10, 1.25) | <0.0001 | 1.16 (1.09, 1.24) | <0.0001 |
| ALS | 1.18 (1.08, 1.30) | 0.0005 | 1.18 (1.09, 1.28) | <0.0001 | 1.18 (1.09, 1.27) | <0.0001 |
| ARS | 1.14 (1.05, 1.24) | 0.002 | 1.14 (1.06, 1.24) | 0.0009 | 1.14 (1.06, 1.24) | 0.0008 |
| CHEW | 1.23 (1.08, 1.40) | 0.001 | 1.24 (1.11, 1.39) | 0.0002 | 1.24 (1.11, 1.38) | 0.0002 |
| CrAS | 1.21 (1.13, 1.29) | <0.0001 | 1.21 (1.13, 1.28) | <0.0001 | 1.20 (1.13, 1.28) | <0.0001 |

ACB denotes Anticholinergic Cognitive Burden, ADS Anticholinergic Drug Scale, ALS Anticholinergic Loading Scale, ARS Anticholinergic Risk Scale, CHEW Chew’s list, CrAS Clinician-rated Anticholinergic Score, and CI confidence interval

# Table S8 Associations between per one-point higher anticholinergic load in a review period and falls in the next review period (residents without dementia)

| Scale | Unadjusted | | Adjusted for age, sex, health condition, and 12 repeated examinations of the association were coded in order as integers from zero to 11 | | Final model, additionally adjusted for the year in which residents entered the facility and time dependent variables | |
| --- | --- | --- | --- | --- | --- | --- |
|  | Odds ratio (95% CI) | p value | Odds ratio (95% CI) | p value | Odds ratio (95% CI) | p value |
| ACB | 0.98 (0.92, 1.05) | 0.56 | 1.00 (0.94, 1.06) | 0.98 | 1.00 (0.94, 1.06) | 0.99 |
| ADS | 1.02 (0.97, 1.08) | 0.40 | 1.04 (0.99, 1.09) | 0.09 | 1.04 (0.99, 1.10) | 0.09 |
| ALS | 1.11 (1.04, 1.18) | 0.0009 | 1.13 (1.07, 1.19) | <0.0001 | 1.13 (1.06, 1.19) | <0.0001 |
| ARS | 1.05 (0.97, 1.14) | 0.26 | 1.06 (0.97, 1.15) | 0.19 | 1.06 (0.97, 1.15) | 0.20 |
| CHEW | 1.09 (1.00, 1.20) | 0.05 | 1.10 (1.00, 1.21) | 0.04 | 1.11 (1.01, 1.21) | 0.03 |
| CrAS | 1.06 (1.01, 1.10) | 0.01 | 1.08 (1.03, 1.13) | 0.001 | 1.08 (1.02, 1.13) | 0.004 |

ACB denotes Anticholinergic Cognitive Burden, ADS Anticholinergic Drug Scale, ALS Anticholinergic Loading Scale, ARS Anticholinergic Risk Scale, CHEW Chew’s list, CrAS Clinician-rated Anticholinergic Score, and CI confidence interval

# Table S9 Associations between per one-point higher anticholinergic load in a review period and falls in the next review period by the year when they entered the facility

| Scale | All residents (n=2300) | | By the year when residents entered the facility | | | |
| --- | --- | --- | --- | --- | --- | --- |
|  | Odds ratio (95% CI) | p value | Year | n | Odds ratio (95% CI) | p value |
| ACB | 1.08 (1.04, 1.12) | <0.0001 | 2014 | 138 | 1.08 (0.88, 1.32) | 0.45 |
|  |  |  | 2015 | 290 | 1.14 (1.05, 1.23) | 0.001 |
|  |  |  | 2016 | 236 | 1.09 (0.93, 1.28) | 0.31 |
|  |  |  | 2017 | 353 | 1.02 (0.92, 1.13) | 0.74 |
|  |  |  | 2018 | 313 | 1.18 (1.05, 1.32) | 0.005 |
|  |  |  | 2019 | 369 | 1.06 (0.95, 1.18) | 0.28 |
|  |  |  | 2020 | 289 | 1.04 (0.94, 1.15) | 0.46 |
|  |  |  | 2021 | 312 | 1.09 (1.00, 1.19) | 0.06 |
| ADS | 1.11 (1.06, 1.15) | <0.0001 | 2014 | 138 | 1.28 (1.07, 1.53) | 0.007 |
|  |  |  | 2015 | 290 | 1.21 (1.08, 1.36) | 0.001 |
|  |  |  | 2016 | 236 | 1.11 (0.91, 1.34) | 0.29 |
|  |  |  | 2017 | 353 | 1.07 (0.99, 1.15) | 0.08 |
|  |  |  | 2018 | 313 | 1.10 (0.96, 1.27) | 0.17 |
|  |  |  | 2019 | 369 | 1.13 (1.00, 1.28) | 0.05 |
|  |  |  | 2020 | 289 | 0.96 (0.83, 1.12) | 0.61 |
|  |  |  | 2021 | 312 | 1.10 (0.96, 1.25) | 0.16 |
| ALS | 1.15 (1.10, 1.21) | <0.0001 | 2014 | 138 | 1.27 (1.06, 1.53) | 0.01 |
|  |  |  | 2015 | 290 | 1.22 (1.04, 1.44) | 0.02 |
|  |  |  | 2016 | 236 | 1.14 (0.94, 1.39) | 0.20 |
|  |  |  | 2017 | 353 | 1.20 (1.06, 1.37) | 0.005 |
|  |  |  | 2018 | 313 | 1.18 (1.02, 1.36) | 0.03 |
|  |  |  | 2019 | 369 | 1.10 (0.94, 1.28) | 0.23 |
|  |  |  | 2020 | 289 | 1.07 (0.93, 1.22) | 0.35 |
|  |  |  | 2021 | 312 | 1.20 (1.08, 1.33) | 0.001 |
| ARS | 1.10 (1.04, 1.17) | 0.002 | 2014 | 138 | 1.36 (0.99, 1.87) | 0.06 |
|  |  |  | 2015 | 290 | 1.20 (1.08, 1.34) | 0.001 |
|  |  |  | 2016 | 236 | 1.15 (0.95, 1.40) | 0.16 |
|  |  |  | 2017 | 353 | 1.00 (0.84, 1.18) | 0.96 |
|  |  |  | 2018 | 313 | 1.22 (1.05, 1.43) | 0.009 |
|  |  |  | 2019 | 369 | 1.06 (0.88, 1.28) | 0.52 |
|  |  |  | 2020 | 289 | 0.98 (0.84, 1.14) | 0.77 |
|  |  |  | 2021 | 312 | 1.14 (0.99, 1.33) | 0.07 |
| CHEW | 1.18 (1.09, 1.27) | <0.0001 | 2014 | 138 | 1.57 (1.14, 2.15) | 0.005 |
|  |  |  | 2015 | 290 | 1.37 (1.21, 1.55) | <0.0001 |
|  |  |  | 2016 | 236 | 1.32 (1.08, 1.61) | 0.008 |
|  |  |  | 2017 | 353 | 1.08 (0.93, 1.26) | 0.30 |
|  |  |  | 2018 | 313 | 1.25 (1.02, 1.55) | 0.03 |
|  |  |  | 2019 | 369 | 1.17 (0.92, 1.49) | 0.20 |
|  |  |  | 2020 | 289 | 1.10 (0.90, 1.34) | 0.34 |
|  |  |  | 2021 | 312 | 1.06 (0.90, 1.24) | 0.48 |
| CrAS | 1.14 (1.10, 1.19) | <0.0001 | 2014 | 138 | 1.20 (0.97, 1.48) | 0.09 |
|  |  |  | 2015 | 290 | 1.25 (1.10, 1.42) | 0.0004 |
|  |  |  | 2016 | 236 | 1.12 (0.96, 1.31) | 0.16 |
|  |  |  | 2017 | 353 | 1.09 (0.95, 1.25) | 0.22 |
|  |  |  | 2018 | 313 | 1.22 (1.10, 1.35) | 0.0002 |
|  |  |  | 2019 | 369 | 1.10 (0.97, 1.24) | 0.13 |
|  |  |  | 2020 | 289 | 1.14 (1.04, 1.24) | 0.004 |
|  |  |  | 2021 | 312 | 1.15 (1.02, 1.31) | 0.03 |

ACB denotes Anticholinergic Cognitive Burden, ADS Anticholinergic Drug Scale, ALS Anticholinergic Loading Scale, ARS Anticholinergic Risk Scale, CHEW Chew’s list, CrAS Clinician-rated Anticholinergic Score, and CI confidence interval.


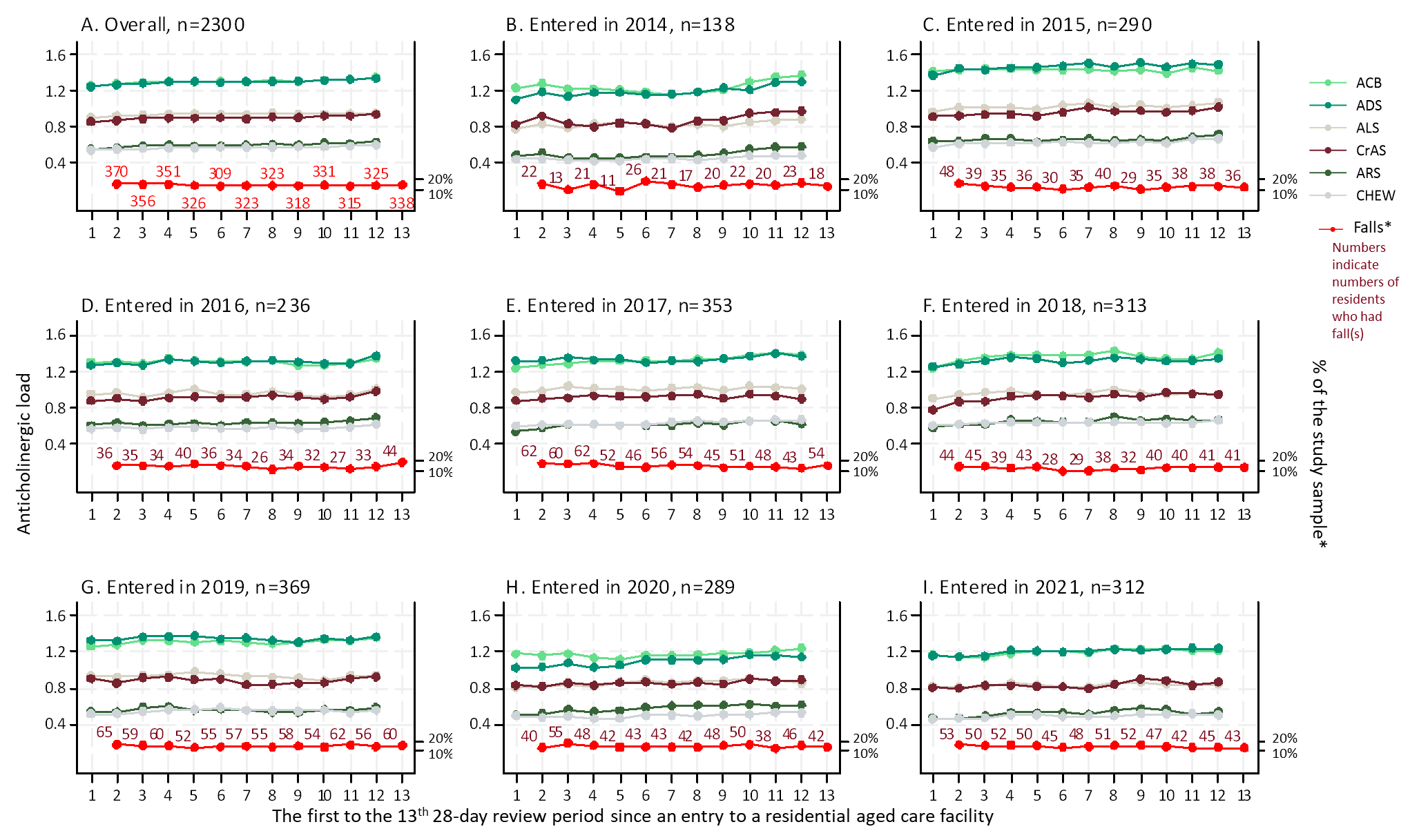


# Figure S1 Anticholinergic load and percentages and numbers of residents who had falls, overall and by the year when they entered the facility
